# PRMT5-dependent p53 escape in tumorigenesis

**DOI:** 10.18632/oncoscience.222

**Published:** 2015-08-31

**Authors:** Yan Li, J. Alan Diehl

**Affiliations:** ^1^ Department of Biochemistry and Molecular Biology, Medical University of South Carolina, Charleston, SC, USA

**Keywords:** p53, arginine methylation, PRMT5, apoptosis, oncogenesis

## Abstract

Extensive studies have characterized mutational disruption of p53 signaling in human cancers. However, the mechanism for bypass of p53 function in tumors retaining wild-type p53 has remained ambiguous. Recent studies suggest that PRMT5, which is frequently elevated in human cancers, cooperates with oncogenic cyclin D1 and leaves marks on p53 by way of arginine methylation, promoting the bypass of wild-type p53, and in doing so, evade apoptosis.

## INTRODUCTION

The tumor suppressor p53 is one of the most extensively studied genes in human disease. More than 50% of solid tumors exhibit missense mutations in the p53 gene, leading to the stable expression of mutant p53 proteins that contribute to malignant progression in as yet poorly understood mechanisms [1]. While solid tumors harbor p53 mutations at high frequency, 80% of human lymphoid malignancies maintain wild-type p53 [2, 3]. Because of the potent tumor suppressive properties of p53, this suggests that alternative mechanisms exist that prevent its tumor suppressive function. Experiments reported by Li et al describe the contribution of PRMT5-dependent methylation of p53 in the bypass of p53-dependent apoptosis [4].

Protein arginine methylation is becoming increasingly recognized as an important modifier of protein function. Arginine methylation is regulated by a family of enzymes referred to as Protein Arginine Methyl Transferases (PRMTs) [5]. PRMTs play pivotal roles in a variety of cellular processes including transcriptional regulation, chromatin regulation, signal transduction and DNA damage repair [6]. One target of significance with regard to cancer biology is p53. The type II protein arginine transferase, PRMT5, interacts with and methylates p53 at R333, R335 and R337 following DNA damage [7]. Mutations that alter these specific arginine residues in p53 have been detected in human cancers and the R337H mutation is associated with cancer risk in a Brazilian family [8], highlighting the potential importance of arginine methylation in the control of p53-mediated events and oncogenesis. However, in vivo models were lacking until the study by Li et al.

Previous investigation of D1-driven malignancy revealed a strong barrier reflecting p53 function. In Em-D1T286A transgenic mice, DNA-damage induced by nuclear D1T286A/CDK4 triggers ATM–CHK2–p53-dependent apoptosis in the premalignant transgenic mice and breach of this barrier could be achieved by genetic deletion of p53 [9,10]. However, mice that develop tumors in this model system retain wild-type p53 at a high frequency [10]. This observation is analogous to hematological malignancies where p53 mutations occur at low frequency and generally late in disease during blast crisis.

**Figure 1:**
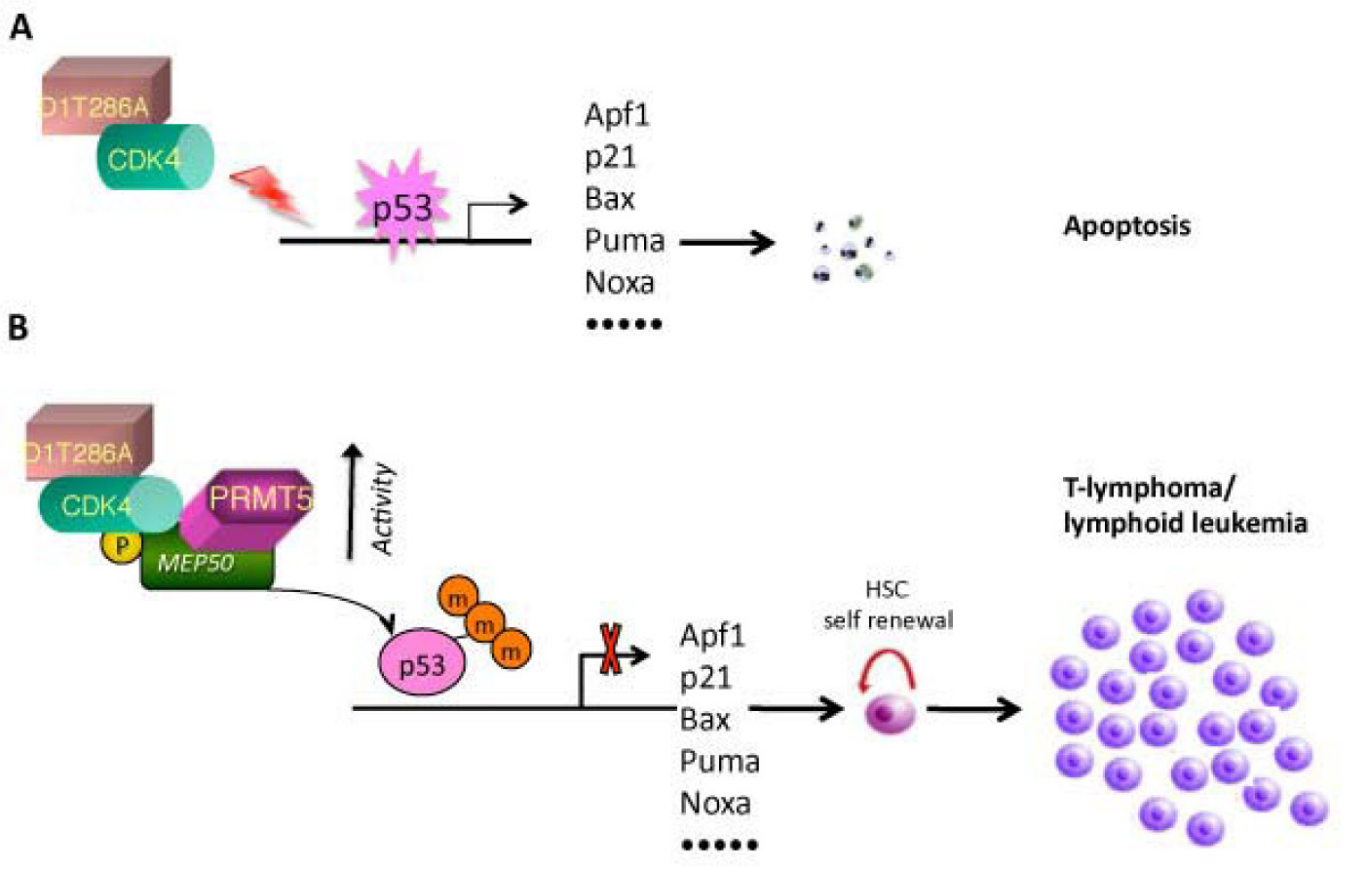
Model of p53 methylation function in tumorigenesis A. p53-dependent transcription of pro-apoptotic genes is activated in response to oncogenic cyclin D1T286A/CDK4. B. In the presence of high level of PRMT5, cyclin D1T286A/CDK4 phosphorylaties MEP50, which in turn catalytically actives PRMT5 on p53 with the consequence of transcriptional inactivation, eventually leading to malignant transformation.

To address mechanisms of p53 bypass during neoplastic development, a bone marrow reconstitution model was used to rapidly establish chimeric, transgenic mice expressing oncogenic cyclin D1 in the hematopoietic lineages. Acute expression of cyclin D1T286A in hematopoietic stem cells (HSC) induced rapid p53-dependent apoptosis resulting in failure of hematopoietic reconstitution [4](Figure 1A). PRMT5 and its co-factor, MEP50, were previously identified as a novel substrate of oncogenic D1T286A/CDK4 [11]. In this previous work, CDK4-dependent phosphorylation of PRMT5/MEP50 complexes was found to increase PRMT5 catalysis contributing to altered gene expression. The current work expands on this theme and demonstrates that PRMT5/MEP50 activation by D1T286A/CDK4 results in high levels of p53 arginine methylation and reduced p53-dependent apoptosis. Strikingly, the authors demonstrated that while infection of HSC with only D1T286A resulted in rapid p53-dependent apoptosis, co-infection with wild-type PRMT5, but not catalytically deficient alleles, abrogated cell death. Reconstitution of lethally irradiated mice with HSC expressing D1T286A and PRMT5 triggered an aggressive T-cell lymphoma that maintained wild-type p53 at 100% frequency (Figure 1B). The ability of PRMT5 to protect and facilitate proliferation of neoplastic cells was abolished when a mutant allele of MEP50 that cannot be phosphorylated by D1T286A/CDK4 (MEP50T5A) was co-transduced. The clinical relevance of this model is emphasized by the demonstration that concurrent elevation of PRMT5 and p53 arginine methylation occurs in primary human cancers such as mantle cell lymphoma and esophageal squamous cell carcinoma, two cancers where cyclin D1 is considered a driver oncogene.

Increased PRMT5 levels have been noted in cell lines derived from a variety of human lymphomas [12,13,14], implying that PRMT5 might function in the context of multiple oncogenic drivers. Consistently, expression of a dominant negative PRMT5 allele or knockdown of endogenous PRMT5 inhibited lymphocyte colony expansion driven by c-Myc, Notch1 and MLL-AF9. These results highlight a potential function for PRMT5 in the context of multiple oncogenic drivers and thus multiple subtypes of non-Hodgkin's lymphoma. These observations demonstrate the strong selection for high levels of PRMT5, the activation of which is indispensible for p53 methylation and tumor pathogenesis. It stands to reason that PRMT5-dependent p53 arginine methylation in cancers is not just a consequence of oncogenesis, but represents a key step in initiating and maintaining this complex process.

Concepts of increasing impact emerge from this study. One concerns the regulation of PRMT5 expression in the context of multiple oncogene-mediated tumors. A second centers on how arginine methylation of histones, E2F1 and p53 (as well as yet to be identified substrates) by PRMT5 is coordinated for neoplastic growth. Finally, is there an arginine-specific demethylase that can reverse this modification? If so since arginine methylation of p53 bypasses the need for p53 mutations, development of a PRMT5 inhibitor could provide a means to reactivate wild-type p53 specifically in tumor cells and rapidly induce tumor cell death.

In summary, the newly validated oncogenic potential of p53 highlights key epigenetic events controlling tumor cell biology in particular to counteract p53 mediated cell death. Targeting p53 methylation pathway in cancer treatment therefore offers a novel strategy for tumors with no p53 mutation.
